# Outdoor air quality data for spatiotemporal analysis and air quality modelling in Ho Chi Minh City, Vietnam: A part of HealthyAir Project

**DOI:** 10.1016/j.dib.2022.108774

**Published:** 2022-11-23

**Authors:** Rajnish Rakholia, Quan Le, Khue Hoang Ngoc Vu, Bang Quoc Ho, Ricardo Simon Carbajo

**Affiliations:** a Ireland's National Centre for Applied Artificial Intelligence (CeADAR), University College Dublin, NexusUCD, Belfield Office Park, Dublin, Ireland; b Institute for Environment and Resources (IER), Ho Chi Minh City 700000, Vietnam; 142 To Hien Thanh St.,Dist.10., HCMC; c Department of Academic Affairs, Vietnam National University, Ho Chi Minh City; Community 6, Linh Trung Ward, HCMC, 700000, Vietnam

**Keywords:** Urban air pollution data, Healthyair, Air quality prediction, Forecasting, Ho Chi Minh City, Vietnam

## Abstract

This article presents outdoor air pollution data acquired from the real-time Air Quality Monitoring Network (AQMN), which was established by the Healthyair project team in Ho Chi Minh City (HCMC), Vietnam. The AQMN is made up of six air pollution monitoring stations spread over the city (Traffic, Residential, and Industrial). Each station measures the same contaminants in the air, including PM_2.5_, TSP, NO_2_, SO_2_, O_3_, CO, and two meteorological factors, temperature and humidity. This data is crucial for air quality modelling, spatiotemporal analysis, correlation analysis, and assessing local air pollution around the city. The data was first obtained in minute frequency, then transformed and produced in hourly frequency for analysis and modelling.

The PM_2.5_ data from this dataset was used to construct an hourly air quality PM_2.5_ forecasting model in the publication titled “AI-based Air Quality PM_2.5_ Forecasting Models for Developing Countries: A Case Study of Ho Chi Minh City, Vietnam” by Rakholia et. al. (2022)

**Specifications Table**

**Table:** 

Subject	Environment Science (air pollution)
Specific subject area	Monitoring urban air pollution using IoT based wireless sensor network.
Type of data	Table data (organized in CSV format)
How the data were acquired	During the first phase of the HealthyAir initiative, six air pollution monitoring stations were established in HCMC. The data were collected from each station and then merged and pre-processed using the Python software program. [2].
Data format	Raw Analyzed (PM_2.5_, NO_2_, CO, SO_2_, O_3_, TSP in µg/m^3^, temperature in°C, relative humidity in %)
Description of data collection	Data was collected from the middle of February 2021 until the middle of June 2022. Six air pollution monitoring stations were installed by the HealthyAir project team in different regions including Traffic, Residential, and Industrial across the city and each of them measures the same number of air pollutants PM_2.5_, NO_2_, CO, SO_2_, O_3_, TSP, and two meteorological parameters Temperature and Humidity. Every minute, each air quality monitoring station communicates the value measured by sensors to a cloud server (data repository). The PM_2.5_ and TSP levels in the air were measured in µg/m^3^, whilst CO, SO_2_, and NO_2_ were recorded in "ppm" and O_3_ was measured in "ppb." The data were transformed to hourly frequency during the data pre-processing step for further analysis and modelling. Data on air contaminants were also converted to the uniform unit (µg/m^3^).
Data source location	Ho Chi Minh City, Vietnam, is the primary source of data. Table 1 shows the location of each station, including longitude and latitude.
Data accessibility	Repository name: Mendeley Data Data identification number [4]: DOI: 10.17632/pk6tzrjks8.1 The data can be downloaded from open access data repository hosted online at https://data.mendeley.com/datasets/pk6tzrjks8/1
Related research article	Rakholia, R., Le, Q., Vu, K., Ho, B. Q., & Carbajo, R. S. (2022). AI-based air quality PM2. 5 forecasting models for developing countries: A case study of Ho Chi Minh City, Vietnam. *Urban Climate*, *46*, 101315. DOI: https://doi.org/10.1016/j.uclim.2022.101315[1]

## Value of the Data

• This is a unique dataset recorded from high-quality sensors network deployed by the HealthyAir project team, which is valuable for understanding and assessing local air quality across multiple regions (traffic, residential, and industrial) in Ho Chi Minh City.
• Data were prepared on an hourly basis, providing sufficient context for future research on air quality assessments, time series modelling, and predictive modelling.
• Since the dataset contains data from numerous air pollutants such as PM2.5, NO2, CO, SO2, O3, and TSP, it can be utilized for correlation analysis, feature selection for air quality modelling, and implementing WHO air quality recommendations [5].
• This dataset can be used to conduct research on determining how air pollution affects human health.
• These data can be useful to researchers interested in spatiotemporal analysis, air quality modelling, and tests on various validation methodologies.
• Researchers can use this data to test various machine learning approaches, and they can be combined with other datasets such as meteorological data or satellite data to estimate air quality.

## 1 Objective

The primary goal of collecting outdoor air quality data was to create a unique dataset that can be used for monitoring regional air quality in the city, developing a policy, assessing the impact of air pollution on human health, and developing solutions to reduce the harmful effects of air pollution on the public in HCMC. This one-of-a-kind dataset was gathered from a real-time air quality monitoring network, allowing for the exploration of numerous issues when constructing machine learning models, devising training procedures, and developing time-series forecasting algorithms. This can benefit researchers working on sustainability, time series analysis, predicting urban air quality, and environmental modelling.

## 2 Data Description

The raw data set comprises 52,549 records gathered between the middle of February 2021 and the middle of June 2022. The raw data contains 52,549 records collected over a period from mid of February 2021 to mid of June 2022. The air quality dataset presented in this article includes date (dd-mm-yyyy HH:00:00), air pollutants such as particulate matter (PM_2.5_), Total Suspended Particles (TSP), Sulfur dioxide (SO_2_), Ozone (O_3_), Nitrogen Dioxide (NO_2_), Carbon Monoxide (CO) in µg/m^3^, and two meteorological parameters Temperature (°C) and Humidity (%), and Station_No includes a number between 1 and 6 that uniquely identifies a station number and its location (Table 1).

**Table 1:** Healthyair air pollution monitoring stations’ information in HCMC.

Station #	Regions	longitude	latitude	Locations
1	Urban background: Industry + Traffic + Residential	10.86994333	106.7960143	Vietnam national university in Ho Chi Minh city, Linh Trung ward, Thu Duc city, HCMC
2	Traffic	10.74097081	106.6171323	20 Nguyễn Trọng Trí street, An Lac ward, Binh Tan district, HCMC
3	Industry	10.81621227	106.6204143	Tan Binh industrial zone/park, Tay Thanh ward, Tan Phu district, HCMC
4	Residential	10.81584553	106.7174282	49 Thanh Da street, Ward 27, Binh Thanh district, HCMC
5	Traffic	10.77636612	106.6878094	268 Nguyen Dinh Chieu street, ward 6, District 3, HCMC
6	Traffic + Residential	10.78047163	106.6594579	MM18 Truong Son street, ward 14, District 10. HCMC

Furthermore, before using this data for analysis and modeling, it is important to understand the data quality: the data was recorded using high-quality sensors, so the records are quite accurate (except outliers at some points due to unforeseen event at random place in the city). There are no duplicates or overlapping values across the dataset, so all records (tuples) are unique. The time component is critical in air quality analysis and modeling; therefore, the entire dataset is prepared in a timely and consistent manner with one-hour intervals and no single timestamp is missing across all stations. Missing values were recorded for some pollutants at some stations, primarily during COVID-19 lockdown periods due to power failures and other uncontrollable factors.

The air quality data from all stations were aggregated and stored into a single file (AirQuality_hcmc.csv), the sample data is shown in Table 2.

**Table 2:** Sample data from air quality dataset.

date	Station_No	TSP	PM2.5	O3	CO	NO2	SO2	Temperature	Humidity
25-01-2022 00:00	4	69.00	30.56	91.84	505.06	92.76	74.86	20.40	12.50
25-01-2022 01:00	4	70.31	31.13	92.36	574.91	92.81	68.12	20.40	12.50
25-01-2022 02:00	4	54.42	24.48	87.09	436.44	77.32	56.77	20.40	12.50
25-01-2022 03:00	4	54.38	24.71	85.39	439.49	76.97	65.06	20.40	12.50
25-01-2022 04:00	4	53.98	24.56	86.11	488.39	78.26	65.94	20.40	12.50

Table 3 shows the statistical summary of air quality data.

**Table 3:** The statistical summary of air quality data across all stations.

Pollutant _Station#	Count	Mean	Std	Min	25%	50%	75%	Max
TSP_1	7835	58.415	33.143	0.000	35.909	52.417	75.081	666.385
TSP_2	9359	45.492	20.136	17.772	36.073	41.240	49.776	1305.860
TSP_3	8456	1.853	9.593	0.000	0.000	0.000	0.000	123.037
TSP_4	9951	64.730	43.706	12.853	37.388	53.668	79.317	938.198
TSP_5	7434	39.289	28.834	0.000	25.467	33.100	47.711	1344.312
TSP_6	9499	47.890	33.674	6.032	26.658	38.753	58.668	657.968
PM2.5_1	7893	20.819	11.860	0.000	13.167	18.538	26.418	301.428
PM2.5_2	9357	19.175	10.642	4.758	12.282	16.091	22.250	95.970
PM2.5_3	8418	23.545	17.657	0.995	12.429	18.363	29.088	207.812
PM2.5_4	9951	26.516	16.885	6.942	16.286	22.533	31.768	403.688
PM2.5_5	7431	15.134	9.262	0.000	9.567	13.063	18.737	290.433
PM2.5_6	9499	20.198	13.466	5.533	12.556	16.758	23.230	310.400
O3_1	2235	99.468	49.941	0.000	64.714	100.080	130.670	307.537
O3_2	9359	113.046	38.133	0.000	90.560	109.601	131.194	372.140
O3_3	8455	77.650	33.061	0.000	56.224	76.132	94.780	349.366
O3_4	9915	85.177	32.145	0.098	68.280	82.479	99.132	377.289
O3_5	2528	98.719	41.639	0.000	76.263	95.402	116.234	332.827
O3_6	9504	97.528	31.579	0.000	77.669	91.427	110.288	300.503
CO_1	7835	810.758	249.644	0.000	717.969	740.068	796.019	3482.155
CO_2	358	1050.802	968.363	0.000	714.579	817.480	1272.442	10809.263
CO_3	8455	922.870	510.827	127.779	605.852	796.470	1098.749	10613.233
CO_4	9951	935.429	488.489	0.000	636.635	836.198	1129.574	6781.837
CO_5	7443	874.389	406.586	115.937	714.015	769.711	877.041	9251.085
CO_6	9504	1360.745	906.670	213.538	716.011	1089.751	1692.260	11551.680
NO2_1	2235	121.361	56.914	0.000	87.560	133.810	164.299	276.654
NO2_2	9359	62.585	26.672	0.000	44.321	68.379	81.930	155.422
NO2_3	8456	61.643	30.323	0.000	39.930	67.689	83.234	257.481
NO2_4	9951	61.286	28.686	0.000	41.153	66.152	82.369	152.254
NO2_5	7442	198.405	105.496	0.000	69.822	272.890	276.152	461.090
NO2_6	9505	112.108	37.894	0.000	94.759	117.343	137.261	554.854
SO2_1	2189	299.807	101.196	7.860	217.460	284.707	368.110	632.293
SO2_2	9350	213.763	87.575	62.007	153.270	190.387	253.267	683.820
SO2_3	8428	196.453	92.029	34.637	133.183	173.684	233.812	689.060
SO2_4	9939	187.382	94.081	2.620	121.830	161.130	235.582	682.073
SO2_5	2490	351.860	115.877	11.034	261.563	336.888	423.567	696.047
SO2_6	9201	249.251	139.018	4.803	137.550	216.150	323.133	699.977

**Fig. 1:**
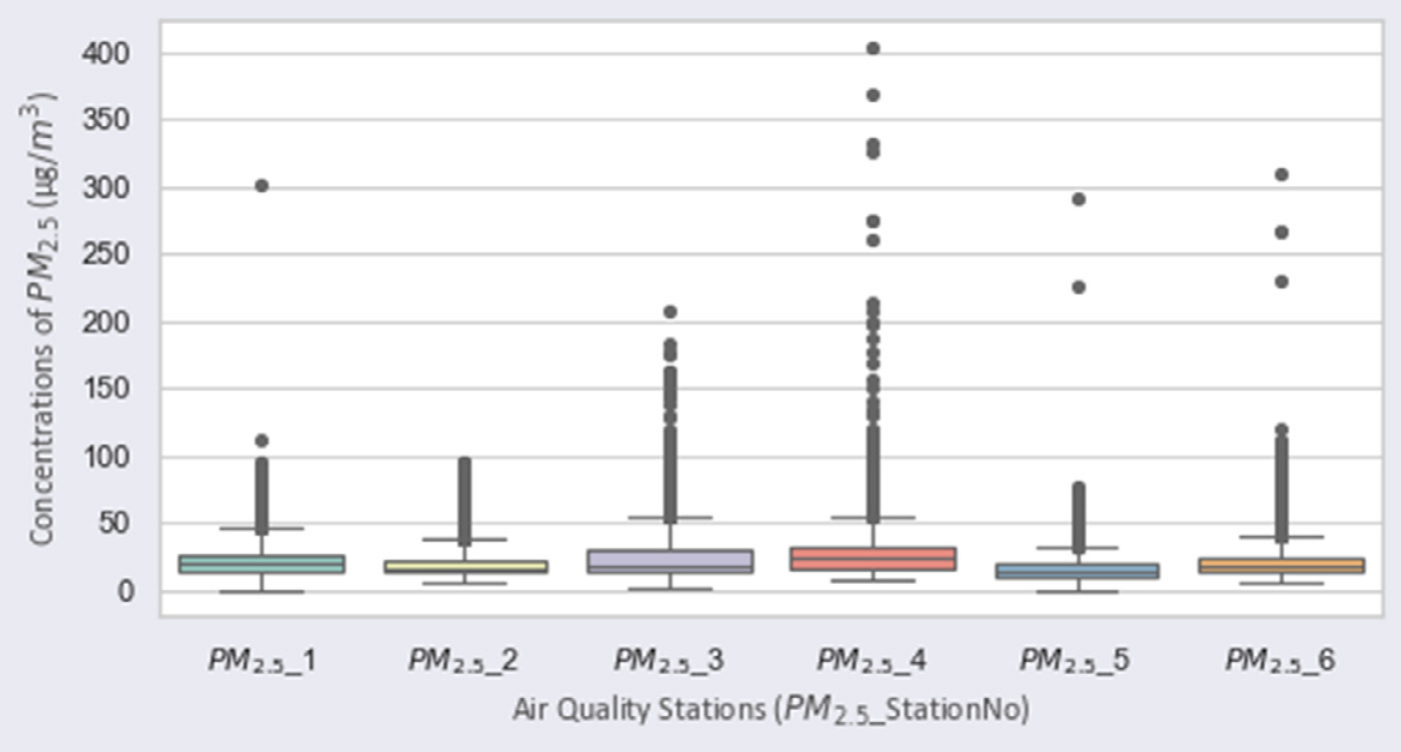
Distribution of PM2.5 over the stations.

**Fig. 2:**
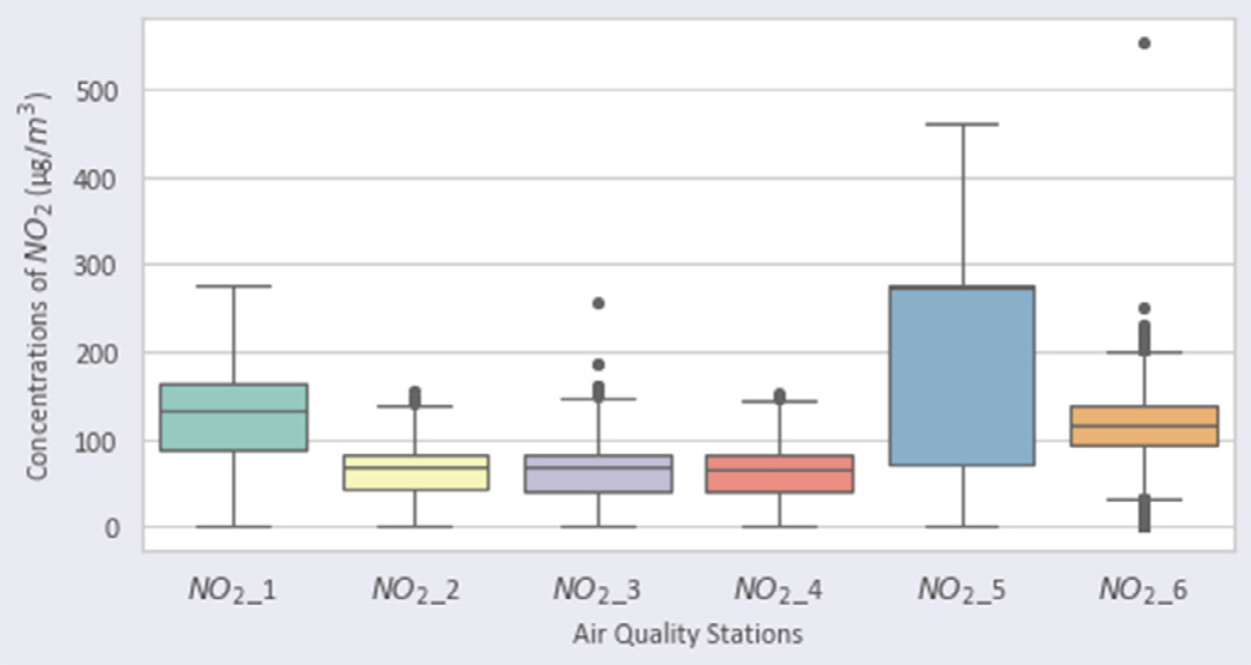
Distribution of NO2 over the stations.

**Fig. 3:**
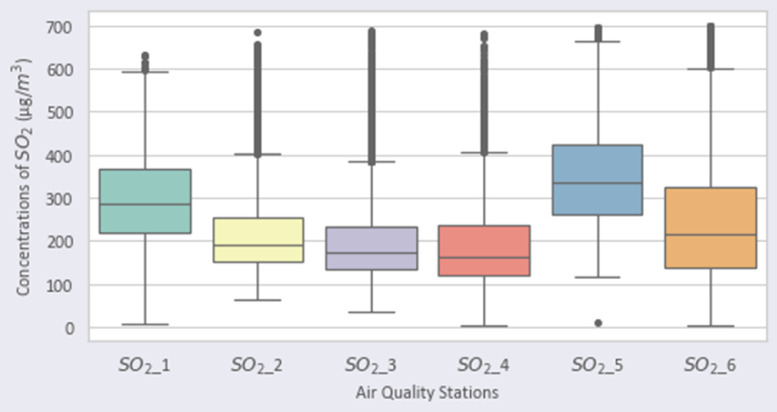
Distribution of SO2 over the stations.

**Fig. 4:**
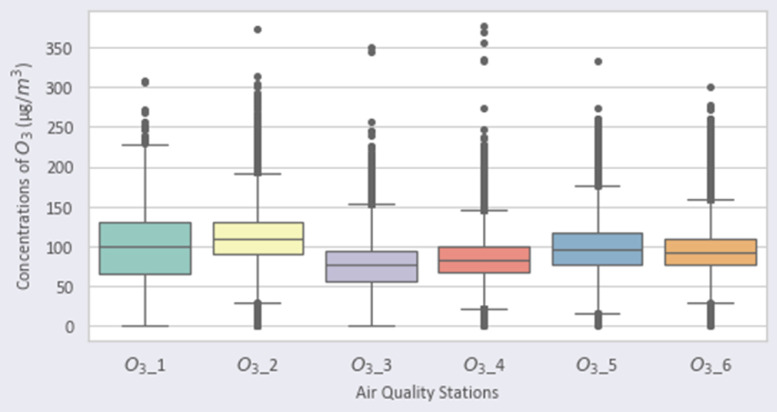
Distribution of O3 over the stations.

**Fig. 5:**
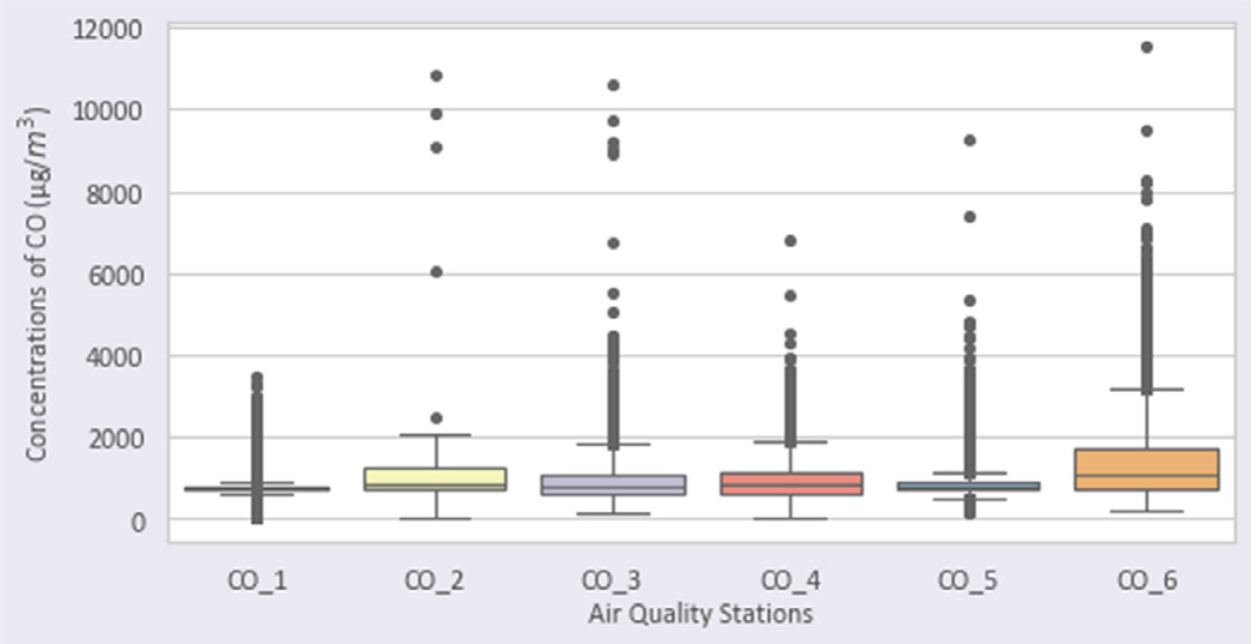
Distribution of CO over the stations.

**Fig. 6:**
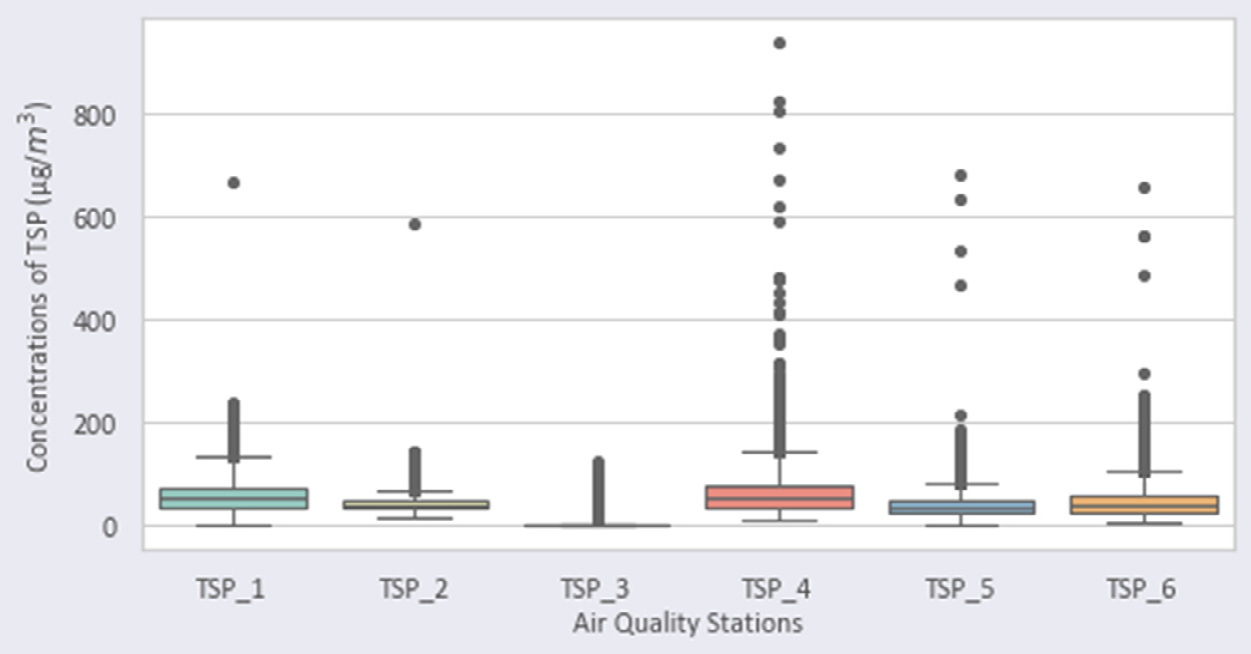
Distribution of TSP over the stations.

Fig. 1, Fig. 2, Fig. 3, Fig. 4, Fig. 5, Fig. 6 show the distribution of air pollutants’ concentrations [3].

## 3 Experimental Design, Materials and Methods

The data presented in this article was gathered from a real-time AQMN comprised of six air pollution monitoring stations. Table 4 describes the technical specifications of the instruments used in the construction of an air pollution monitoring station.

**Table 4:** Technical specifications of air quality monitoring instrument

Product Name	Outdoor air quality analyzer
Model	PM SCAN
Dimensions	220mm (H) x 160mm (W) x 450mm (Depth)
Description	This device can monitor, store and transmit data on its own in the web server storage
Output	RS 232/485, USB, Wifi, Ethernet
Power	Powered by a 220V AC adapter
Other information	Waterproof
Producer Source	Sensoronic Co.,Ltd, Korea

**Fig. 7:**
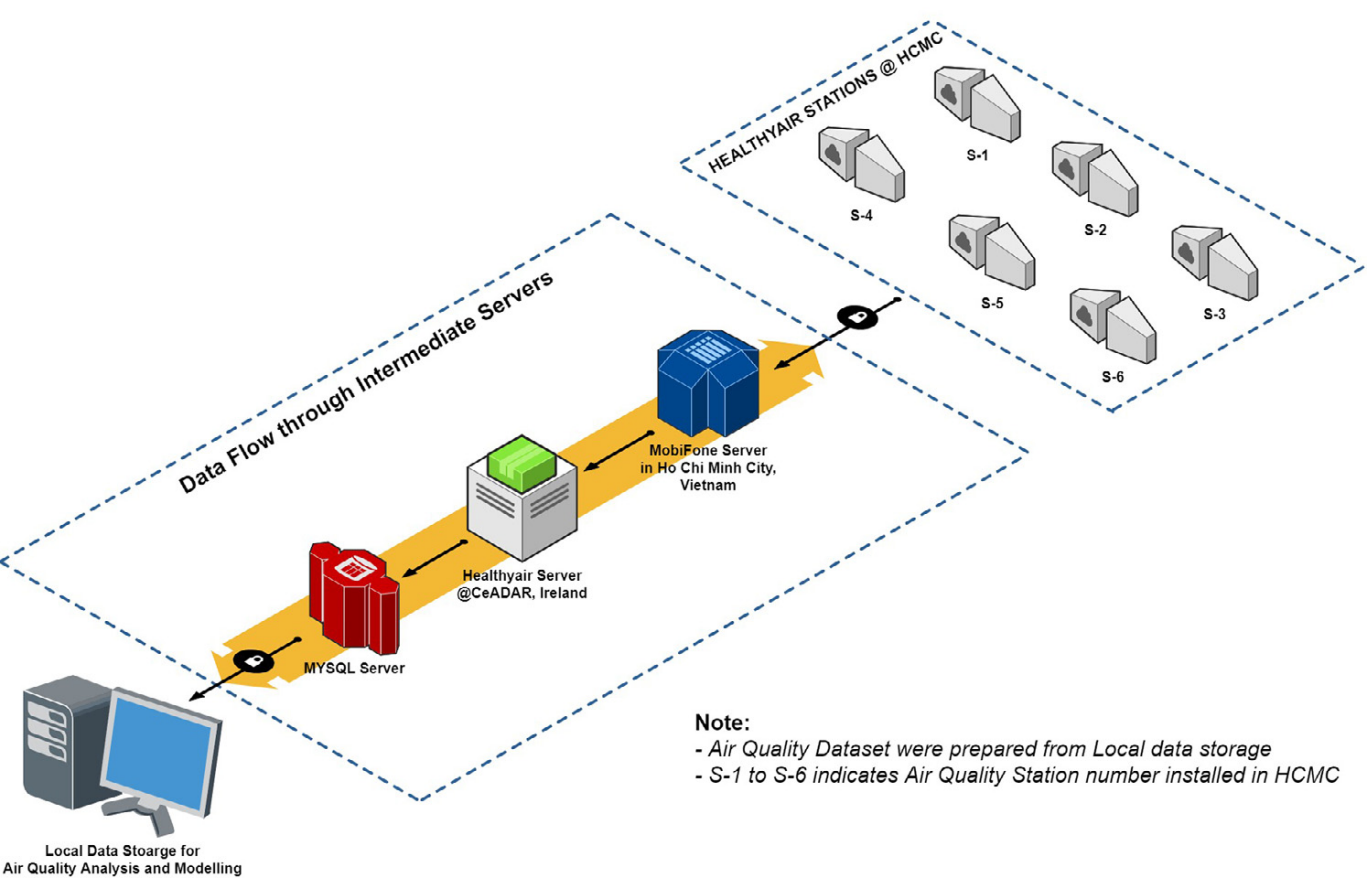
Air quality data acquiring flow.

The locations of air quality stations in HCMC were chosen with the goal of monitoring air quality in a variety of places, including traffic, urban background, residential areas, industrial districts, and high population density.  Every 60 seconds, all stations measured the identical set of air pollutant concentrations, which were then relayed to a cloud server (Fig. 7). Each station's data for each day was saved on the server in a separate (.csv) file. Following that, all csv files were imported into a Python workspace for merging and re-sampled on an hourly basis.

Following that, all negative values were removed from the dataset since the sensors occasionally recorded exaggerated amounts of air contaminants. All negative values were replaced with ‘nan,' and that was treated as missing values in the dataset [2].

Originally, air pollutants PM_2.5_ and TSP were measured in µg/m^3^ at Healthyair stations, whilst CO, SO_2_, NO_2_, and O_3_ were measured in ``ppm'' and ``ppb'' respectively. Table 5 shows the calibration rate for converting air quality concentrations from ``ppm'' and ``ppb'' to uniform unit µg/m^3^.

**Table 5:** Calibration rate used for converting into (µg/m^3^).

#	Parameters	Units	Calibration rate to Convert into µg/m^3^
1	CO	ppm	1146
2	O_3_	ppb	1.963
3	NO_2_	ppm	1882
4	SO_2_	ppm	2620
5	TSP, PM_2.5_ (Already in µg/m^3^, no need to convert it)		

The data was then saved on a MySQL server, which allows users to retrieve, sort, search, and filter the data using SQL queries for air quality study, modelling or further analysis. Finally, we exported the data from the MySQL database in csv format.

## Ethics Statements

There were no ethical requirements for data collection and processing, and this study did not involve animal or human investigations.

## CRediT authorship contribution statement

**Rajnish Rakholia:** Data curation, Software, Writing – original draft. **Quan Le:** Supervision, Writing – review & editing, Validation. **Khue Hoang Ngoc Vu:** Visualization, Investigation. **Bang Quoc Ho:** Project administration, Conceptualization, Funding acquisition, Writing – review & editing. **Ricardo Simon Carbajo:** Project administration, Conceptualization, Methodology, Funding acquisition.

## Declaration of Competing Interest

The authors declare that they have no known competing financial interests or personal relationships that could have appeared to influence the work reported in this paper.

## Appendix Supplementary materials

Image, application 1

Image, application 2
